# A predictive Bayesian approach to risk analysis in health care

**DOI:** 10.1186/1471-2288-7-38

**Published:** 2007-08-23

**Authors:** Terje Aven, Karianne Eidesen

**Affiliations:** 1 Department of industrial economics, risk management and planning, University of Stavanger, Stavanger, Norway

## Abstract

**Background:**

The Bayesian approach is now widely recognised as a proper framework for analysing risk in health care. However, the traditional text-book Bayesian approach is in many cases difficult to implement, as it is based on abstract concepts and modelling.

**Methods:**

The essential points of the risk analyses conducted according to the predictive Bayesian approach are identification of observable quantities, prediction and uncertainty assessments of these quantities, using all the relevant information. The risk analysis summarizes the knowledge and lack of knowledge concerning critical operations and other activities, and give in this way a basis for making rational decisions.

**Results:**

It is shown that Bayesian risk analysis can be significantly simplified and made more accessible compared to the traditional text-book Bayesian approach by focusing on predictions of observable quantities and performing uncertainty assessments of these quantities using subjective probabilities.

**Conclusion:**

The predictive Bayesian approach provides a framework for ensuring quality of risk analysis. The approach acknowledges that risk cannot be adequately described and evaluated simply by reference to summarising probabilities. Risk is defined by the combination of possible consequences and associated uncertainties.

## Background

To analyse risk in health care, the Bayesian approach is widely acknowledged as a proper framework, see e.g. [1] Spiegelhalter et al. (2003) and the references therein. However, implementing the Bayesian approach in a practical context is difficult. The standard text-book Bayesian analysis ([2] Bernardo and Smith 1994, [3] Singpurwalla 2006) introduces fictional parameters that are difficult to understand and they complicate the analysis. To explain this in more detail, consider a Probabilistic Risk Analysis (PRA) for a specific operation at a specific hospital. Let *p *be a parameter expressing the probability that the operation is unsuccessful, i.e. an accidental event occurs. To determine *p *we use models such as event trees and fault trees. Formalising this means that *p *is computed using a function *f *of a set of parameters *q*, i.e. *p *= *f*(*q*). Here *q *is a vector and could include parameters expressing for example human error probabilities and probabilities of failures of safety barriers. The function *f *is a model, a representation of the relationship between the "true" parameters *p *and *q*.

Using the formula *p *= *f*(*q*) and expressing uncertainty distributions of the parameters *q*, we can establish an uncertainty distribution of *p*. This is in practice normally carried out by means of Monte Carlo simulation. If data become available, Bayesian updating can be carried out using Bayes' theorem. In this way the prior distribution of *p *is converted to the posterior distribution of *p*. The approach is referred as the probability of frequency approach ([4] Kaplan and Garrick 1981).

The parameters *p *and *q *reflect stochastic uncertainties (aleatory uncertainties). Subjective probabilities are assigned to reflect epistemic uncertainties; what are the correct parameter values? When data become available, the epistemic uncertainties will be reduced ([3] Singpurwalla 2006).

This approach to risk analysis has in our view three main weaknesses:

1. Focus is on fictional parameters which are difficult to understand.

2. The message of the analysis is being disturbed by a discussion of uncertainties in parameters constructed by the analysts.

3. The analysis is too complex to be implemented in practice.

To understand the meaning of the parameters we have to introduce infinite populations of similar situations. The parameter *p *is a mind constructed quantity not existing in real life, interpreted as the proportion of unsuccessful operations when considering an infinite number of similar operations. But what is the meaning of this population? What is "similar"? Do we cover operations in other hospitals, in other countries, and using different procedures? If we are to assess uncertainties of average performance of quantities of this population, it is essential that we have clarified its meaning.

Since the parameters are unknown, we have to address uncertainties. We do this by the standard Bayesian updating approach as mentioned above, and we are led to the posterior distributions of the parameters. A full implementation of this approach in a PRA could typically mean hundreds of parameter distributions, and the result being very wide credibility intervals for the output parameters. What is then the message from the analysis? The uncertainties are so large that we cannot conclude on the risk level and the effect of risk reducing measures.

In our view the probability of frequency approach creates uncertainties, not inherent in the system being studied, by introducing fictional parameters and then become uncertain about their values.

To reduce the uncertainties, it may be tempting to allow for uncertainty distributions just on some of the input parameters, and reflect some but not all factors causing uncertainties. We often see such a reduced analysis in practice. But what is then the meaning of the output probability distributions? The analysis is in fact incomplete. Such a simplified uncertainty analysis could also be motivated by the fact that a full uncertainty analysis along these lines is extremely resource demanding and time-consuming. It is certainly relevant to question the cost-effectiveness of performing such analyses. The analyses produce a vast number of figures and distributions, but what do they add to the decision-making process? We refer to discussions in [5] Aven (2003) and [6] Aven and Kvaløy (2002).

In our view the probability of frequency approach cannot be justified, in general and for health care applications in particular. To cite [7] Marx and Slonim (2003),

"Healthcare is in many ways different from other industries. It depends upon human interaction between patient and a practitioner during illness and recovery. This interaction is emotional, significant, and some would argue, essential for recovery. However, it is "humanness" in health care that is also responsible for some of the safety problems. Practitioners are not computers and have a limited ability to process multiple pieces of often-contradictory information. Practitioners need to eat, drink, sleep and have bathroom breaks. They also have personal lives and stresses that may alter their focus or influence their attentions while they are caring for patients. These "human factors" are important considerations when mapping patient safety problems. The ability to include these "sociotechnical" effects into the PRA model improves its use as a tool to facilitate patient safety interventions".

For such a setting, is it meaningful to search for the correct risk numbers? Certainly not. Hard data showing the truth about risk will not be available. Instead we should consider risk analysis as a tool for systematising the available knowledge and uncertainties. Such information will be useful for supporting decisions, as it is based on the best available knowledge.

This leads us to the predictive Bayesian approach, focusing on predictions and uncertainty assessments of observable quantities ([8] Barlow 1998, [5] Aven 2003). We believe that such a predictive approach is more appropriate for analysing risk, in general and for the health care area in particular, as there is only one level of uncertainty, stemming from lack of knowledge. The purpose of the present paper is to present and discuss concepts and principles for analysing risk using such an approach in the health care area. Our main focus is probabilistic risk analysis (PRA).

PRAs are used to analyse complex systems where there is a lack of data to accurately predict the future performance of the system. Insights are obtained by decomposing the system into subsystems/components for which more information is available. Overall failure probability and risk is a function of the system's architecture, and of the probabilities of failure of the different subsystems/components ([9] Paté-Cornell and Dillon 2001).

PRAs are used in many industries to support decision-making in complex situations involving high risks, see e.g. [10] Bedford and Cooke (2001). Such analyses are also seen in health care and patient safety ([7] Marx and Slonim 2003, [11] Battles and Kanki 2004), and in the paper we specifically address their use in this area.

[7] Marx and Slonim (2003) and [11] Battles and Kanki (2004) study an application of PRA, the ST-PRA (sociotechnical probabilistic risk assessment). The ST-PRA specifically addresses human error and behavioural dimensions. Note that the standard PRA is not restricted to technical issues. Although the traditional emphasis in PRAs has been technical issues, human factors have been incorporated in PRAs for many years. A component in the system may be a human error. Recently we have also seen incorporation of organisational issues in PRAs, cf. the I-Risk ([12] Papazoglou et al. 2003), ARAMIS ([13] Duijm et al. 2004; [14] Dujim and Goossens, 2006), the BORA projects ([15] Aven et al. 2006) and the SAM approach ([16] Paté-Cornell & Murphy 1996).

Such analyses must in our view be based on the use of subjective probabilities. It is considered meaningless to search for accurate estimates of some "true" risk as in the probability of frequency approach, reflecting the average state of a number of human and organisational factors.

The terminology used in this paper related to risk analyses is in line with the ISO standard on risk management terminology, [17] ISO (2002). A risk analysis includes identification of hazards and threats, cause analyses, consequence analyses and risk description. The results of the analyses are then evaluated. The totality of the analyses and the evaluations are referred to as risk assessments.

The paper is organised as follows. First we present and discuss a simple example to illustrate some of the basic features of the predictive Bayesian approach. Then we address another example where we show the difference between the predictive Bayesian approach and the more traditional text-book Bayesian analysis. Based on these examples we summarise our main points and conclude.

## Methods

### The predictive Bayesian approach. A simple PRA health care application

In the predictive Bayesian approach there are no fictional parameters introduced, and no reference to true probabilities. The focus is on the observable quantities, and the actual population. The observable quantities are quantities that express states of the "world"; quantities that are unknown at the time of the analysis but will, if the system/activity actually is implemented, take some value in the future and possibly become known.

**Figure 1:**
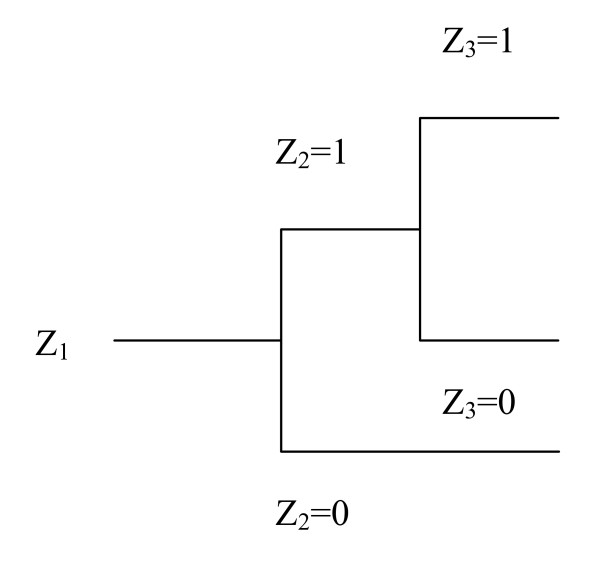
An event tree example.

Applying the predictive approach, we may for example focus on the number of accidental events *X *in some specified operations. We introduce no fictional parameters. By modelling (for example using event trees and fault trees) we establish a link *g *between *X *and observables on a more detailed system level, denoted *Z *= (*Z*_1_, *Z*_2_, ..., *Z*_m_). Here *Z*_i _could be the number of hazardous situations of a certain type occurring during some operations or an indicator function which equals 1 if a specific safety barrier fails and 0 otherwise. To illustrate, consider the simple event tree model in Figure 1. In this model *Z*_1 _equals the number of hazardous situations occurring, *Z*_2 _is equal to 1 if the first safety barrier fails and 0 otherwise, and *Z*_3 _is equal to 1 if the second safety barrier fails and 0 otherwise. The model *g *is defined by

*X *= *g*(*Z*) = *Z*_1_·*Z*_2_·*Z*_3_

as *X *is given by the number of hazardous situations where both safety barriers fail.

Note that in practice we seldom express the *g *function explicitly as in this example. The function is implicitly given by the system representation, for example the event tree, the fault tree or the influence diagram ([5] Aven 2003, [8] Barlow 1998).

The quantities *X *and *Z *are unknown and in the analysis we predict these quantities and express associated uncertainties. First we assess the uncertainties of *Z*. This is used by assigning subjective probabilities according to the Bayesian paradigm, and through *g *we establish a probability distribution of *X*. This probability distribution *P*(*X *≤ *x*) is conditional on the assessor's background information *K*, and we write *P*(*X *≤ *x *| *K*). The model *g *is a part of this background information. Consequently there is no meaning in speaking about model uncertainty. Of course, we may address the accuracy of the model, but that is another issue. Poor accuracy of the model does not produce uncertainties in the assigned probability of *X*, as the probability is conditional on the use of this model. This is in contrast to the probability of frequency approach where model uncertainty exists and should be addressed.

Returning to the event tree example, we establish a prediction of *X *by the following reasoning: General statistical data show that the hazardous situation of this type typical occurs 20 times, and hence we obtain the prediction *Z*_1_* = 20 of *Z*_1_. To assess the first safety barrier performance, consider 10 hazardous situations. Say that the assessor predict 1 failure. Then this gives aprobability *P*(*Z*_2 _= 1) = 0.1. Now given that the hazardous situation occurs and the first safety barrier fails, how reliable is the second safety barrier? Say that we assign a probability of 0.4 in this case. That means that we predict 4 failures out of 10 cases. Or we could simply say that 0.4 reflects the assessor's uncertainty. The reference is a certain standard such as drawing a ball from an urn. If we assign a probability of 0.4 for an event *A*, we compare our uncertainty of *A *to occur with drawing a red ball from an urn having 10 balls where 4 are red.

Hence *P*(*Z*_2 _= 1 | *Z*_1 _= 1) = 0.4, and using the model *g*, we obtain the prediction X* = *Z*_1_*·*Z*_2_*·*Z*_3_* = 20·0.1·0.4 = 0.8.

We predict 1 accidental event for these operations. Next we need to address the associated uncertainties. One way of doing this is to specify a 90% prediction interval for *Z*_1_. If the interval is [10, 100], the assessor is 90% certain that the number of hazardous situations *Z*_1 _will be in this interval, given the background information of the analysis. The interval is determined based on the available information, that is, relevant data and expert judgments. From this analysis we obtain a 90% prediction interval for *X *equal to [0, 4], using the same probabilities for the barrier safety failures as above.

More generally we may express a probability distribution for *Z*_1_, and from this we obtain a distribution for *X*. From a practical point of view the distribution of *Z*_1 _could be based on intervals for the number of hazardous situations and accidental events, such as [0, 5], [6, 10], [11, 15], [16, 20], etc. and associated probability assignments for each of these intervals.

There is no need for introducing a Bayesian updating procedure for a case like this. All relevant information is incorporated into the probability assignments for the quantities *Z*_i _and *X *– no updating is required. For a comment on this, see the discussion and conclusion section.

A parametric probability distribution could also be used to express the analysts' uncertainties, for *Z*_1 _and *X*. However, care has to be shown when it comes to the choice of distribution class and the interpretation of the distribution and its parameters. In our case, one may feel that a natural distribution choice for the number of hazardous situations *Z*_1 _is to use the Poisson distribution, with a specified parameter value. But this distribution has a rather small variance (equal to its mean) and it may therefore not be appropriate for describing the analysts' uncertainty. Instead a gamma-Poisson distribution (negative binomial distribution) ([8] Barlow 1998) could be used as this distribution has a larger variance. Note that there is no correct distribution. The distribution chosen is a subjective assignment describing the analysts' uncertainties. Nonetheless, we should always question if the assignments are reasonable given the background information. If there are large uncertainties about the number of hazardous situations, it will be hard to justify the use of the Poisson distribution. If the background information is strong, the Poisson distribution may be more adequate ([5] Aven 2003, p. 81).

In practice many events studied in PRA are rare, and the associated prediction would be no occurrences. The uncertainties would then simply be expressed through the probability for the event to occur.

In this framework there is only one type of uncertainties stemming from lack of information (epistemic uncertainties). This idea is sometimes questioned. It is felt that some probabilities are easy to assign and feel sure about, others are vague and it is doubtful that the single number, say 0.1 in the above analysis, means anything. Should not the vagueness be specified?

To respond on this, let us remember that a probability *P*(*A*) is in fact a short version of a conditional probability of *A *given the background information *K*, i.e. *P*(*A*) = *P*(*A*|*K*). This means that even if we assign the same probability for two probabilities, they may be considered different as the background information is different. In some cases we may know much about the process and phenomena leading to the event *A*, and in some cases very little, but still we assign a probability of say 0.50 in both cases. However, by considering several similar events of the type *A*, as in our event tree example, i.e. we change the performance measure, the difference in the background information will often be revealed. An illustrating example of this is given by [18] Lindley (1985), p. 112. Thus care has to be shown when defining the performance measures, and when evaluating probabilities in a decision-making context. We always need to address the background information, as it provides a basis for the evaluation.

A probability is in our context a subjective measure of uncertainty. However, subjective probabilities are often defined using utilities ([3] Singpurwalla 2006). This is in our view unfortunate, as a probability should not be linked to our attitude to winning or loosing money, but to uncertainty.

## Results

### Comparison of the predictive Bayesian approach and the traditional text-book Bayesian approach

Consider a patient (L) suffering from a specific disease and faced with the following treatment options:

Treatment A. This is a traditional treatment with a moderate high success rate, and the complications are rather few. The treatment is however not able to give the patient full recovery.

Treatment B: This is an alternative new treatment, with the potential of giving the patient full recovery. The experiences with this treatment is not strong, but the performance records from the hospital H offering this treatment are promising.

The patient L is subject to a choice between these two treatments. Choosing what risks to take is not a medical decision ([19] Schneider 2006). As a basis for the patient's decision, a risk picture is presented. In the following we will present this picture using a traditional text-book Bayesian approach and the predictive Bayesian approach.

### Traditional text-book Bayesian analysis

Let p_1 _denote the probability that treatment A results in success for an arbitrary patient. Similar we define p_2 _for treatment B. We interpret these probabilities as success rates in an infinite population of patients, undergoing the same treatment. These probabilities (parameters) are unknown and prior distributions are established describing the assessor's uncertainties before observations. When data become available, these distributions are updated by the standard Bayes updating procedure to give the posterior distributions for the parameters. Probability models are introduced to carry out this updating procedure. Best estimates of the probabilities are provided, using the mean of the posterior distributions. Similar analyses are performed for the complication rates.

These posterior distributions are presented to the patient, and constitute the patient's basis for making his/her choice.

### Predictive Bayesian approach

In this case, the patient asks for the following information:

• The historical statistical data, i.e. success rates for various defined populations.

• Risk analysis performed by experts, providing predictions and assessing uncertainties related to the following quantities:

o Proportion of successful treatments for patients at the hospital H

o Successful treatment of patient L.

The risk analyses are carried out according to the approach outlined in the previous section. Expert judgments are included, to incorporate all relevant information about the treatments as well as the patient. The reported results are summarised below:

Consider 100 patients at the hospital H, and let D be the proportion of successful treatments.

Treatment A: Predicts D equal to 90,

90% prediction interval for D: [85, 95]

P(successful treatment for patient L) = 0.90

Treatment B: Predicts D equal to 95,

90% prediction interval for D: [70, 99]

P(successful treatment for patient L) = 0.97

General risk influencing factors, i.e. factors that can influence the risk and hence the outcome:

• Hospital H infrastructure for performing the treatments

• Physician's competence level

• General health condition and medical history of the patient

A discussion of these factors and possible others that could cause unsuccessful results is carried out.

The above risk picture provides the basis for the patient's decision. Using the new treatment the expressed risk is lower, when we look at the probability of successful treatment. This new treatment also has the potential of giving the patient full recovery. However, the experience basis for this treatment is also lower. This creates some uncertainty, which has to be balanced against the lower computed risk numbers.

## Discussion and conclusion

The main features of the traditional text-book Bayesian approach and the predictive Bayesian approach is summarised in Table 1, using the dimensions theoretical perspective and application oriented perspective:

**Table 1:** Summary of main features of the traditional text-book Bayesian approach and the predictive Bayesian approach

***Traditional text-book Bayesian approach***		***Predictive Bayesian approach***	
*Theoretical perspective*	*Application oriented perspective*	*Theoretical perspective*	*Application oriented perspective*

Focus on fictional parameters	Average patient performance	Focus on observable quantities	Performance of the individual or group considered
Prior and posterior distributions	The experts' uncertainties about the unknown parameters	Prior and posterior distributions	The experts' uncertainties about the observable quantities
There exists underlying true probabilities	Probabilities and risks are unknown and need to be estimated	Probability is a subjective measure of uncertainty, conditional on the assessor's background information	Probability is assigned by the assessor
Model uncertainty exists	Model uncertainty needs to be covered by the analysis	Model uncertainty does not exist.	The accuracy of the models needs to be addressed

The differences are demonstrated by the above example. For this example, we consider the predictive approach to produce the most informative risk picture, as it is the patient perspective that is of concern. Data showing the historical average performance of the treatments obviously are interesting for the patient, but the assessment including the specific knowledge about the hospital H and him/her as a patient, is likely to be given most weight.

It may be responded that it is also possible to produce predictive distributions in the traditional text-book Bayesian approach by taking the expectations of the p_i_s. Yes, that is true. However, such a distribution includes the assessor's uncertainties of the p_i_s. Why should we incorporate this uncertainty when our ambition is to assess uncertainties about successful treatment of patient L. We have to be careful in defining what are relevant populations. If we shift to the hospital or national level, would we conclude in the same way? Yes, the key quantity of interest is:

D: the proportion of successful treatments among those to be carried out the coming years

and not an underlying theoretical probability p expressing that treatment A results in success for an arbitrary patient. By introducing such a probability, we introduce a limiting quantity, a fictional element, which leads to the wrong focus, accurate estimation of p instead of D.

According to the Bayesian perspective, probability is a subjective measure of uncertainty ([20] Lindley 2000). Probabilities are used to express uncertainties about unknown quantities. However, we have to acknowledge that uncertainties about observables cannot be adequately described and evaluated simply by reference to summarising probabilities. There is a need for seeing beyond these values. Computed probabilities are subjective assignments conditioned on the background information (including assumptions and suppositions). The probabilities are not objective values. The analysis could produce poor predictions. The risk picture has to include aspects related to uncertainties in phenomena and processes. Surprises may occur and by just addressing probabilities such surprises may be overlooked.

We are of course not able to predict all surprises – if that had been the case, they would not have been surprises. The risk perspective should be broad enough to allow the uncertainties and possible surprises to be an important part of the overall risk picture.

A search procedure needs to be established to identify the uncertainty factors. Such a procedure can be based on an initial analysis addressing historical records and expert judgments. The risk influencing factors mentioned above also indicate areas that may be of concern. Such could be reflected in the background information of the assigned probabilities, or calculation procedures could be developed which more explicitly take them into account, see e.g. [15] Aven et al. (2006). Furthermore, the assumptions and suppositions of the probability assignments provide an additional checklist. We would also like to draw attention to the list of special consequence features presented by [21] Renn and Klinke (2002) (see also [22] Kristensen et.al. 2006). Examples of such features are:

a) Delay effects – which describe the time of latency between an initial event and the actual damage.

b) Reversibility – which describe the possibility to restore the situation to the state before damage occurred.

This feature classification system can be used as a checklist for ensuring the right focus of the analysis, i.e. that we address the appropriate consequences attributes. But it can also be used as a checklist for identifying relevant uncertainty factors. For example, the feature "delay effects" could lead to a focus on activities or mechanisms that could initiate deteriorating processes causing future surprises.

Addressing the uncertainties also mean to consider the *manageability*; i.e. to what extent is it possible to control and reduce the uncertainties, and obtain desirable outcomes? Some risks are more manageable than others, meaning that the potential for reducing the risk is larger for some risks compared to others. By proper uncertainty management, we seek to obtain desirable consequences.

Expressing risk, also means to perform sensitivity analyses. The purpose of these analyses is to show how sensitive the output risk indices are with respect to changes in basic input quantities, for example assumptions and suppositions.

Risk is described by addressing such issues along with the probabilities. It gives in our view a sound basis for risk analysis in general and for the health care in particular. Such a broad perspective on risk is in line with the following definition of risk:

Risk related to an activity is defined by the combination of the possible consequences of the activity and associated uncertainties ([23] Aven and Kristensen 2005). Subjective probabilities are used to assess the uncertainties.

Risk analyses are tools providing insights about risks. But they are just tools – they have limitations. Their results are conditioned on a number of assumptions and suppositions. The analyses are not expressing objective results. We should not put more emphasis on the predictions and assessments of the analyses than what can be justified by the methods being used. Nonetheless, risk analyses could be useful as a decision supporting tool in situations with large uncertainties. They summarize the knowledge and lack of knowledge concerning critical operations and other activities, and give in this way a basis for making rational decisions.

To ensure high quality risk analyses we believe that the following points, among others, should be highlighted ([24] Aven 2004)

- The analysis team has a thorough understanding of the system performance and the decision-making process and context.

- Models used are sufficient accurate representations of the world, their goodness in describing the world have been evaluated.

- All observable quantities of the analysis are precisely defined.

- The meanings of risk and uncertainty are fully understood and consistently treated.

- The background information for the analyses is documented.

Adopting the predictive Bayesian approach is no guarantee for meeting all these requirements. However, it provides a framework for ensuring quality of the analyses along these lines. Risk analyses of health operations, such as PRAs, must in our view be based on the use of subjective probabilities. The probabilistic analyses need to be based on modelling and use of expert judgments, and the probabilities should express uncertainties in events and other real quantities, given the available information.

The essential points of the analyses are identification of observable quantities, prediction and uncertainty assessments of these quantities, using all the relevant information. Bayesian updating procedures are of less importance, as seen from the examples above. The Bayesian updating procedure may be used for incorporating new information, but its applicability is in many cases rather limited. In practice we will often not perform a formal Bayesian updating to incorporate new observations – rethinking of the whole information basis and approach to modelling is required when we conduct the analysis at a particular point in time.

Of course, there are situations where such procedures provide a useful basis of the analysis. As an example, consider an operation where we are concerned about a quantity *U *expressing the health condition of the patient. The quantity *U *is unknown and continuous measurements *V*_1_, *V*_2_, ..., provide information about *U*. Then starting from a prior distribution of *U*, we establish a posterior distribution of *U *using the measurements *V*_1_, *V*_2_, .... We then need a distribution of *V *given *U*, reflecting the accuracy of the measurements. Such a procedure is consistent with the predictive Bayesian approach as all quantities introduced are observables.

For a successful implementation of risk analysis in health care, we need an assignment process which is simple, that works in practice for the number of probabilities and probability distributions to be determined. We should not introduce distribution classes with unknown parameters when not required. Furthermore, meaningful interpretations must be given to the distributions classes and the parameters whenever they are used. There is no point in speaking about uncertainty of parameters unless they are observables, i.e. not fictional.

## Competing interests

The author(s) declare that they have no competing interests.

## Authors' contributions

The authors have contributed equally to this work. Both authors have read and approved the final manuscript.

## Pre-publication history

The pre-publication history for this paper can be accessed here:


